# Acute Mycophenolate Mofetil Overdose Managed Conservatively

**DOI:** 10.7759/cureus.17417

**Published:** 2021-08-24

**Authors:** Prabhat Sharma, Ashish Guragain, Sunil Adhikari, Subash Dahal, Bibek Dhungana

**Affiliations:** 1 Internal Medicine, Shankarapur Hospital, Kathmandu, NPL; 2 Internal Medicine, Manipal College of Medical Sciences, Pokhara, NPL; 3 General Practice and Emergency, Patan Academy of Health Sciences, Lalitpur, NPL; 4 Internal Medicine, Nobel Medical College, Biratnagar, NPL; 5 Internal Medicine, KIST Medical College, Lalitpur, NPL

**Keywords:** adverse effects, mycophenolate mofetil, mycophenolic acid, overdose, poisoning, toxicity

## Abstract

Mycophenolate mofetil (MMF) is an immunosuppressant drug widely used in post-transplant patients and the treatment of various inflammatory conditions. It is considered a relatively safe drug with minimal adverse effects. We managed an acute overdose of 19 grams (g) of MMF with a suicidal intention in a 17-year-old female with no significant past medical history. Apart from episodes of mild headaches, she did not develop other symptoms, laboratory abnormalities, or complications.

## Introduction

Mycophenolate mofetil (MMF) is an immunosuppressant drug affecting the proliferation of T and B lymphocytes. It inhibits the enzyme inosine-5′-monophosphate dehydrogenase in the de-novo production of guanosine nucleotides [1]. Within a therapeutic range, MMF is considered a relatively safe drug compared to other immunosuppressive drugs [2]. The commonly observed adverse effects associated with the long-term use of MMF include gastrointestinal symptoms and dose-related bone marrow suppression [2,3]. Diarrhea, nausea, vomiting, infections, fatigue, headache, cough, leukopenia, and anemia are commonly reported. Less frequent adverse effects include esophagitis, gastritis, gastrointestinal tract hemorrhage, and invasive cytomegalovirus infection [2,3]. However, there is a paucity of literature describing the acute toxicity of MMF [3]. Here, we report the case of a 17-year-old female who ingested 19 g (38 tablets of 500 mg each) MMF tablets (Mycophen®-500) with suicidal intent. She was managed conservatively in a secondary care hospital in Nepal. The patient and her family members confirmed the ingestion of MMF; further verified by the evidence of empty blister packs of the drug brought by them. Although there are reports of acute MMF overdose in individuals who were taking it regularly as per their prescription; this report describes an acute MMF overdose in an individual who had never taken MMF in the past. To the best of our knowledge, this is the first case of an acute MMF overdose reported in Nepal.

## Case presentation

A 17-year-old female presented to the emergency room with an alleged history of ingestion of multiple drugs, including 38 tablets of MMF 500 mg and five tablets of phenobarbital 60 mg four hours earlier. She consumed her mother’s medicines with suicidal intention. According to her mother, she was found in an intoxicated and ataxic state with slurring of speech two hours before the presentation at the emergency room. After that, she developed drowsiness and was brought to the hospital. Her past medical and psychiatric history were unremarkable. Upon arrival, she was in a drowsy state. Her heart rate was 115 beats per minute (bpm) and her blood pressure (BP) was 110/70 millimeters of mercury. Her respiratory rate was 18 per minute with symmetrical chest rise and oxygen saturation was 97%. Neurological examination revealed ataxia and slurred speech but she could follow commands. She had grossly intact cranial nerve functions, normal motor strength, and reflexes. Other systemic examination findings were unremarkable. Her body mass index (BMI) was 34.8 kg/m^2^. Her management in the emergency room consisted of intravenous hydration; continuous monitoring of her condition and vital signs. She was fully alert, her heart rate was 80 bpm, and her BP was 120/70 mmHg within four hours of arrival. After that, she did not complain of any other symptoms.

On admission, her laboratory studies showed a white blood cell (WBC) count of 7,800/mm^3^, hemoglobin of 12.2 g/dL, and platelet count of 287,000/mm^3^. Blood chemistry revealed serum creatinine of 1.2 mg/dL, aspartate aminotransferase of 21 IU/L, alanine aminotransferase of 19 IU/L, and glucose of 80 mg/dL. She had normal urinalysis, prothrombin time-international normalized ratio (PT-INR), electrolytes, and arterial blood gases. Chest x-ray and electrocardiogram were unremarkable. Ultrasonography of the abdomen did not reveal any significant abnormalities except fatty liver. From day 2 of hospitalization, she reported occasional mild headaches relieved by acetaminophen. Otherwise, her hospital stay was uneventful. She was monitored and managed conservatively in the Intensive Care Unit (ICU) for two days and later in the general medical ward for the next three days. After a psychiatric evaluation, she was diagnosed with an unspecified mood disorder with suicidal ideation. She was prescribed aripiprazole and advised for a regular follow-up. She was discharged on the fifth day of admission. Her laboratory studies were last done on day 4, which demonstrated a WBC count of 6,500/mm^3^, hemoglobin of 12.5 g/dL, platelet count of 263,000/mm^3^, serum creatinine of 0.9 mg/dL, aspartate aminotransferase of 13 IU/L, alanine aminotransferase of 15 IU/L, and glucose of 95 mg/dL. The electrolyte levels, PT-INR, and urinalysis were within normal parameters. Her laboratory studies during the time of admission and before discharge are summarized in Table 1.

**Table 1:** Laboratory studies of the patient ALP: Alkaline phosphatase; AST: Aspartate aminotransferase; ALT: Alanine transaminase

Blood analysis	Day 1	Day 4	Reference range
Hemoglobin	12.2	12.5	11.0-16.0 g/dL
White blood cells	7,800	6,500	4,000-11,000 cells/mm^3^
Neutrophils	76	70	40%-75%
Lymphocytes	20	26	25%-45%
Platelets	287,000	263,000	150,000-450,000 cells/mm^3^
Blood sugar (random)	80	95	60-140 mg/dL
Urea	27	30	8-45 mg/dL
Creatinine	1.2	0.9	0.4-1.4 mg/dL
Sodium	137	138	135-146 mEq/L
Potassium	3.7	3.7	3.5-5.0 mEq/L
Total bilirubin	0.8	0.9	0.4-1.4 mg/dL
Direct bilirubin	0.2	0.3	0.1-0.4 mg/dL
ALP	201	159	64-306 IU/L
AST	19	15	5-37 IU/L
ALT	21	13	8-42 IU/L

## Discussion

MMF is widely used in the treatment of post-transplant patients, systemic lupus erythematosus, and other several inflammatory conditions [2]. The commonly reported side effects of its long-term use are gastrointestinal (nausea, dyspepsia, vomiting, and diarrhea) and hematological (leukopenia, anemia, and thrombocytopenia) [2], but the effects of acute overdose are less reported. Our patient consumed a high dose of MMF (19 g) at once along with 300 mg of phenobarbital. The patient’s acute presentation of ataxia, slurring of speech, and an intoxicated state are the well-described effects of phenobarbital [4]. Since these are not the commonly described effects of MMF toxicity, we did not attribute them to it.

The MMF level in blood is often monitored by measuring the level of mycophenolic acid (MPA), the de-esterified and active moiety of MMF [5]. The terminal half-life of the MPA is very short (2.6 h to 7.4 h), and decontamination by gastric lavage using activated charcoal is not considered mandatory [3], so gastric lavage was not performed in our patient. Even though gastric lavage was not performed, we did not observe the incidence of severe adverse events in her. It is shown that the MPA levels-toxicity relationship is not well established, and no correlation has been found between MPA levels and the adverse effects of MMF [5,6]. Therefore, MPA levels are rarely monitored [3], and it was not performed in our patients either.

The existing literature on acute MMF overdose shows that most of the patients remained asymptomatic. Based on the systematic analysis of all reported acute overdoses from the Swiss Toxicological Information Center (STIC) as well as a literature review of MPA overdose cases from 1995 to 2013, most of the acute poisoning cases were a result of attempted suicide in adults, whereas children were subject to accidental poisoning [3,7]. A few of them developed self-limiting and mild symptoms like headache, nausea, vomiting, dizziness, and abdominal pain with a favorable outcome and none had any severe complications [3]. No symptoms and complications were reported after acute ingestion of 10 g of MMF in a 24-year-old patient who was taking the drug regularly for lupus nephritis [8]. Similar was the case in a 17-year-old female with granulomatosis with polyangiitis, who ingested 20 g of MMF at once [9]. A case report by Doi et al. reported seizures, leukopenia, thrombocytopenia, and acute renal failure in a 17-year-old girl with lupus nephritis who took 1 g of MMF daily for one week. But her symptoms resolved in a few weeks without complications after the discontinuation of the drug [10]. In a 40-year-old female kidney recipient under maintenance dose of MMF, acute ingestion of 25 g of MMF resulted in moderate leukopenia [11].

We acknowledge a few limitations in the management of this case. MPA levels monitoring could have been performed to establish a correlation between MPA levels in blood and toxicity if she had developed significant adverse effects. The initial symptoms were assumed to be due to phenobarbital toxicity. Therefore, the early clinical manifestations of MMF overdose could have been masked.

## Conclusions

Hence, from our observation of this case and other similar studies, MMF is a relatively safe drug considering acute overdose. An acute overdose of 38 times the most frequently prescribed dose did not have any severe consequences except for mild headaches. Further studies and case reports reporting similar overdose with only MMF and overdose in those who are not on regular MMF prescriptions are necessary to provide more information.
